# A Rushan Acid Whey-Derived *Limosilactobacillus fermentum* A001-A-08 Attenuates Lipid Accumulation in High-Fat-Diet-Induced Zebrafish

**DOI:** 10.3390/foods15142536

**Published:** 2026-07-17

**Authors:** Hao Chen, Siyu Li, Shiwei Zhao, Luxin Miao, Wenjun Wu, Jiayi Mi, Xiufang Dong, Yan Jiang

**Affiliations:** 1 School of Public Health, Dali University, 22 Wanhua Road Dali Bai Autonomous Prefecture, Dali 671000, China; ch_chenhao_2001@163.com (H.C.); 17842262243@163.com (S.L.);; 2 Dali Lesson Dairy Co., Ltd., Dali 671000, China

**Keywords:** Rushan acid whey, *Limosilactobacillus fermentum*, *lactic acid bacteria*, probiotic candidate, preliminary safety evaluation, zebrafish, lipid metabolism

## Abstract

Rushan acid whey is an underutilized by-product of a traditional fermented dairy food and may contain probiotic lactic acid bacteria. This study screened cholesterol-lowering strains from Rushan acid whey and evaluated *Limosilactobacillus fermentum* A001-A-08 for functional traits, preliminary safety, and lipid-lowering potential in high-fat-diet-induced zebrafish. A001-A-08 showed cholesterol removal, bile salt hydrolase activity, acid/bile/simulated gastrointestinal tolerance, auto-aggregation, gamma-hemolysis, and a mostly susceptible antibiotic profile. A 10^6^ CFU/mL bath concentration maintained larval survival, heart rate, and morphology and enabled intestinal localization during the observation period. In high-fat zebrafish, A001-A-08 reduced Oil Red O-stained lipid accumulation, improved hepatic morphology, lowered TC and TG, and increased HDL-C. Transcriptomic, metabolomic, qRT-PCR, and 16S rRNA sequencing analyses indicated that these phenotypic changes were associated with alterations in lipid transport, lipoprotein assembly, bile acid-related metabolism, selected metabolite features, and gut microbial composition. These findings support A001-A-08 as a promising Rushan acid whey-derived probiotic candidate, while genome-level safety assessment, mammalian oral-administration studies, and food-matrix validation remain necessary before functional-food application.

## 1. Introduction

Dyslipidemia and excessive lipid deposition are major metabolic disturbances associated with fatty liver disease, obesity, and cardiovascular risk [1,2]. Therefore, lipid metabolism is an important target for evaluating food-derived microbial interventions, especially candidate LAB strains that may modulate cholesterol handling, bile acid metabolism, lipoprotein assembly, and host–microbe metabolic interactions. Probiotic lactic acid bacteria (LAB) have attracted attention as food-compatible microbial resources that may contribute to lipid regulation and functional fermented-food development [3,4]. Because probiotic effects are strain-specific, each candidate strain requires functional characterization and preliminary safety evaluation before food application [5,6].

Traditional fermented foods and their by-products are important reservoirs for discovering autochthonous LAB with probiotic, safety-related, and techno-functional properties [7,8]. Rushan is a traditional acid-coagulated dairy product consumed in Yunnan Province, China. During Rushan manufacture, acid whey is generated as a nutrient-rich liquid by-product containing lactose, organic acids, peptides, minerals, and microorganisms adapted to the acidic dairy-fermentation environment. Although this by-product is often underutilized, it represents a valuable ecological source for isolating autochthonous LAB with potential functional and safety-related traits.

Cholesterol removal and bile salt hydrolase (BSH) activity are commonly used as early indicators for screening cholesterol-lowering LAB, although these in vitro assays cannot alone prove physiological efficacy [9,10,11]. In vivo models are therefore needed to determine whether candidate strains attenuate lipid accumulation and metabolic disturbance [12,13]. Zebrafish larvae provide a convenient vertebrate model for lipid metabolism studies because they develop rapidly, are optically transparent, require small sample volumes, and allow direct visualization of whole-body lipid accumulation using Oil Red O staining. The study was designed to support A001-A-08 as a probiotic LAB candidate that requires further genome-level safety assessment, mammalian validation, and food-matrix testing. The present results should be interpreted as preliminary in vivo evidence of lipid-lowering potential, not direct proof of functional-food efficacy [14,15,16].

In this study, nine LAB isolates from Rushan acid whey were screened for cholesterol-lowering traits, gastrointestinal tolerance, auto-aggregation, hemolysis, and antibiotic susceptibility. The main contribution of the work lies in connecting this traditional dairy by-product-derived strain resource with zebrafish-based preliminary in vivo evaluation and integrated transcriptomic, metabolomic, qRT-PCR, and gut microbiota profiling. *L. fermentum* A001-A-08 was selected and further evaluated in high-fat-diet-induced zebrafish. Transcriptomic, broad-coverage metabolomic, qRT-PCR, and 16S rRNA gene amplicon sequencing was used to describe host and microbial changes accompanying the lipid-lowering phenotype. The study was designed to support A001-A-08 as a probiotic LAB candidate that requires further genome-level safety assessment, mammalian validation, and food-matrix testing.

## 2. Materials and Methods

### 2.1. Bacterial Strains, Media, Reagents, and Instruments

Nine LAB isolates derived from Rushan acid whey were used in this study (Table S1 Bacterial strains used in this study, E023-A-05 (*Lactiplantibacillus plantarum*), A001-A-08 (*Limosilactobacillus fermentum*), B097-A-07 (*Lacticaseibacillus paracasei*), C179-A-05 (*Lactiplantibacillus plantarum*), C179-D-08 (*Lactiplantibacillus plantarum*), H035-A-11 (*Lacticaseibacillus paracasei*), C133-B-14 (*Enterococcus faecium*), D158-A-04 (*Enterococcus durans*), H066-A-03 (*Lacticaseibacillus paracasei*)). MRS broth was used for routine cultivation, and its composition is listed in Table S4. The main reagents and instruments used for biochemical, staining, culture, and microscopic analyses (HDL-C, LDL-C, TC, TG (Nanjing Jiancheng Bioengineering Institute, Nanjing, China), Oil Red O staining kit (Servicebio Technology Co., Ltd., Wuhan, China) are listed in Tables S2 and S3.

### 2.2. In Vitro Functional and Safety-Related Characterization

Acid tolerance was evaluated in MRS broth adjusted to pH 2.0, 2.5, and 3.0. Bile salt tolerance was evaluated at 0.03%, 0.15%, and 0.30% bile salt. Simulated gastric and intestinal fluid tolerance was assessed after gastric fluid exposure for 3 h and intestinal fluid exposure for 4 and 8 h. Viable counts were expressed as log CFU/mL.

Cell-surface auto-aggregation was measured at 2, 4, 24, and 48 h. Cholesterol removal was determined using a colorimetric assay. BSH activity was evaluated using bile-salt-containing indicator agar. Hemolytic activity was assessed on blood agar, and antibiotic susceptibility was interpreted according to Clinical and Laboratory Standards Institute criteria [17]. Because whole-genome sequencing was not performed at this stage, this safety assessment was considered preliminary and could not directly evaluate virulence genes, mobile genetic elements, plasmids, or transferable antibiotic-resistance determinants.

### 2.3. Identification and Preparation of A001-A-08

The selected strain (A001-A-08) was characterized by colony morphology and Gram staining. The sequence was compared against public databases for taxonomic identification. A freeze-dried A001-A-08 powder was prepared using skim milk powder and trehalose as protective agents. Its viable counts in sterile water and E3 medium were measured before zebrafish exposure experiments (Table S6).

### 2.4. Zebrafish Ethics, Concentration Selection, and Intestinal Localization

All zebrafish procedures were reviewed and approved by the Life Sciences Ethics Committee of Dali University (approval No. LSECDU-2026002). Zebrafish larvae (*Danio rerio*) were used to establish a high-fat-diet-induced lipid metabolism disorder model. Rearing conditions were controlled, water quality was monitored regularly, and fish were humanely euthanized after anesthesia at the end of the experiment in accordance with the approved protocol. E3 medium was prepared as listed in Table S5.

A preliminary bath-exposure experiment tested A001-A-08 at 10^5^–10^9^ CFU/mL. Survival rate, malformation rate, heart rate, and gross morphology were used to select the working concentration. CM-DiI labeling was used to visualize intestinal localization during the observation period. This assay was interpreted as evidence of short-term retention rather than stable long-term colonization.

### 2.5. High-Fat Zebrafish Model and Lipid Phenotype Assays

Control, high-fat model, and A001-A-08 intervention groups were established. Whole-body Oil Red O staining was used to evaluate neutral lipid deposition. H&E staining of sagittal sections was performed to assess hepatic morphology. TC, TG, HDL-C, and LDL-C levels were measured using commercial kits listed in the Supplementary Table S2.

### 2.6. RNA Sequencing and qRT-PCR Validation

RNA sequencing was conducted using biological replicates from the control, model, and A001-A-08 groups (*n* = 3). Raw reads were quality-filtered by removing reads containing 3′-adapter sequences and reads with average quality scores below Q20. Sequencing quality was evaluated using clean-read yield, Q20/Q30 percentages, GC content, base-quality distribution, mapping statistics, and sample-correlation analysis. Differentially expressed genes were identified using a DESeq2-based workflow with thresholds of *p* < 0.05 and |log2 FC| > 1 [18]. GO and KEGG enrichment analyses were performed to identify enriched biological processes and pathways [19,20,21]. Nine lipid metabolism- and transport-related genes were selected for qRT-PCR validation: *apoba*, *apoa4b.2*, *mttp*, *cyp8b1*, *cideb*, *acot16*, *abcc2*, *slc26a3.2*, and *fabp10a*. Relative expression was calculated using the 2^−ΔΔCt^ method [22,23].

### 2.7. Broad-Coverage LC-MS Metabolomic Profiling

Broad-coverage LC-MS metabolomic profiling was conducted for the three experimental groups (*n* = 3 per group). Raw LC-MS data were converted to mzXML format using MSConvert in ProteoWizard (v3.0.8789) and processed in XCMS (v3.12.0) for peak detection, retention-time correction, and alignment using key parameters including ppm = 15, peakwidth = c (5, 30), mzdiff = 0.01, and method = cent Wave. Peak areas were normalized by total peak area to reduce systematic variation. Metabolite annotation was performed using public and in-house spectral resources, including HMDB, MassBank, LipidMaps, mzCloud, KEGG, and an in-house standard database, and 1963 MS/MS-level metabolites were annotated [20,24,25,26]. PCA, OPLS-DA, differential metabolite screening, heatmap visualization, and KEGG pathway mapping were performed. Differential metabolite features were screened using *p* < 0.05 and VIP > 1.

### 2.8. 16S rRNA Gene Amplicon Sequencing

Gut microbial communities were analyzed by 16S rRNA gene amplicon sequencing. Primer- and adapter-filtered reads were denoised, merged, and chimera-filtered using DADA2, and ASVs were taxonomically assigned using SILVA 138.1 and QIIME2 resources [27,28,29]. Across the nine samples, DADA2 input reads ranged from 65,201 to 167,476, and final non-chimeric reads ranged from 56,886 to 130,473, with a mean of 86,205 reads per sample. A total of 1339 ASVs were initially inferred, and 1189 ASVs remained after filtering mitochondrial/chloroplast-associated assignments and other non-target features. Alpha diversity, beta diversity, taxonomic composition, and LEfSe biomarkers were analyzed [30,31]. PICRUSt2 functional prediction was interpreted as exploratory and hypothesis-generating.

### 2.9. Statistical Analysis

Data are presented as mean ± SD unless otherwise stated. One-way analysis of variance followed by post hoc comparison was used when assumptions were satisfied. Non-parametric tests were applied where appropriate. *p* < 0.05 was considered statistically significant.

## 3. Results

### 3.1. Functional Screening, Preliminary Safety Assessment, Concentration Selection, and Intestinal Localization of A001-A-08

**Figure 1:**
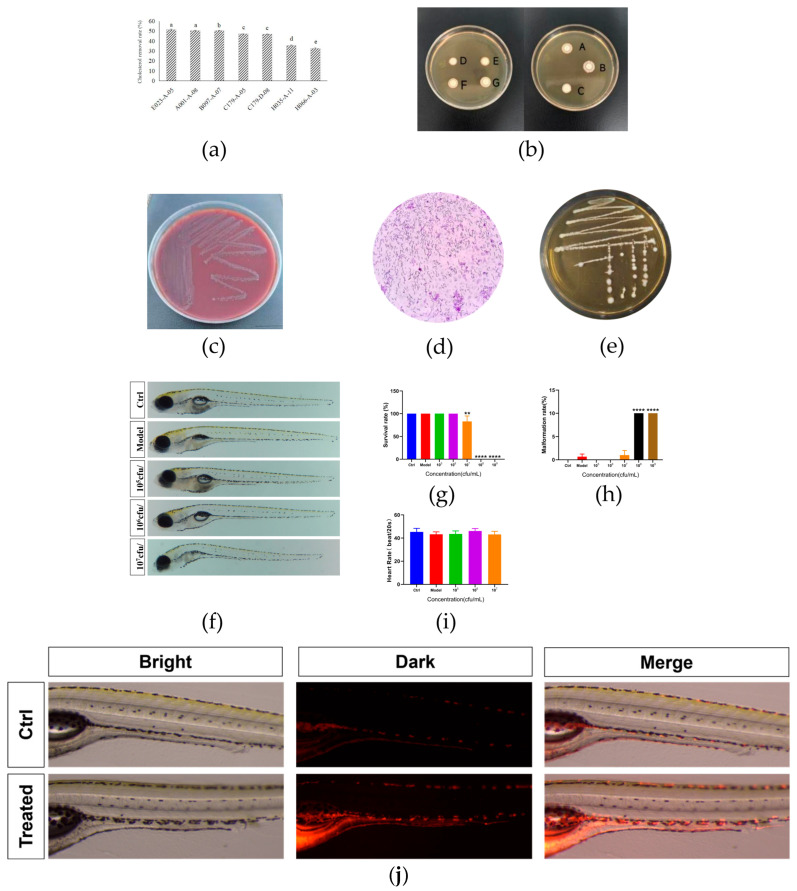
Functional screening, preliminary safety assessment, concentration selection, and intestinal localization of *L. fermentum* A001-A-08. (**a**) In vitro cholesterol removal by Rushan-derived LAB isolates. (**b**) Representative BSH indicator plates, A(A001-A-08); B(E023-A-05); C(H035-A-11); D(H066-A-03); E(C179-D-08); F(C179-A-05); G(B097-A-07). (**c**) Hemolysis result of A001-A-08. (**d**) Gram staining. (**e**) Colony morphology. (**f**) Zebrafish morphology under different A001-A-08 concentrations (10^5^–10^9^ CFU/mL). (**g**) Survival rate. (**h**) Malformation rate. (**i**) Heart rate. (**j**) CM-DiI fluorescence showing intestinal localization during the observation period. Data in quantitative panels are presented as mean ± SD. Different lowercase letters above bars indicate significant differences among strains (*p* < 0.05). For zebrafish concentration-screening panels, asterisks indicate significant differences compared with the control group as indicated in the graph (** *p* < 0.01, **** *p* < 0.0001).

The cholesterol-removal assay showed strain-dependent differences among Rushan-derived LAB isolates (Figure 1a). The final candidate strain was selected through a stepwise screening strategy based on comprehensive performance across cholesterol removal, bile salt hydrolase activity, acid and bile tolerance, simulated gastrointestinal-fluid tolerance, auto-aggregation ability, hemolytic safety, and antibiotic susceptibility, and Limosilactobacillus fermentum A001-A-08 showed the most suitable overall profile among the nine Rushan acid whey-derived LAB isolates. A001-A-08 showed a cholesterol-removal rate of 50.95 ± 0.27%, and it was retained for subsequent experiments. Representative BSH indicator plates showed precipitation zones for isolates with BSH activity (Figure 1b). Additional screening outputs, including the cholesterol standard curve (y = 0.0162x − 0.0127 R^2^ = 0.9979), hydrophobicity (A001-A-08-08, B097-A-07, C179-A-05, H066-A-03, H035-A-11, E023-A-05, C179-D-08), and complete hemolysis-related plates (*L. rhamnosus*, A001-A-08-08, B097-A-07, C179-A-05, H066-A-03, H035-A-11, E023-A-05, C179-D-08), are shown in Figure S1.

A001-A-08 showed γ-hemolysis on blood agar (Figure 1c), Gram-positive rod-shaped morphology (Figure 1d), and colony morphology consistent with LAB on MRS agar (Figure 1e). The complete acid-tolerance, bile-salt-tolerance, simulated gastrointestinal-fluid-tolerance, auto-aggregation, and antibiotic-susceptibility results are listed in Tables S7–S11.

In the zebrafish concentration-screening assay, 10^5^ and 10^6^ CFU/mL maintained larval morphology similar to the control and model groups, whereas higher concentrations produced more adverse responses (Figure 1f–i). Based on survival rate, malformation rate, heart rate, and morphology, 10^6^ CFU/mL was used as the working concentration. CM-DiI fluorescence was detected in the intestinal region during the observation period, indicating intestinal localization or short-term retention of A001-A-08 (Figure 1j).

### 3.2. A001-A-08 Attenuated Lipid Accumulation and Improved Biochemical Lipid Indices in High-Fat Zebrafish

**Figure 2:**
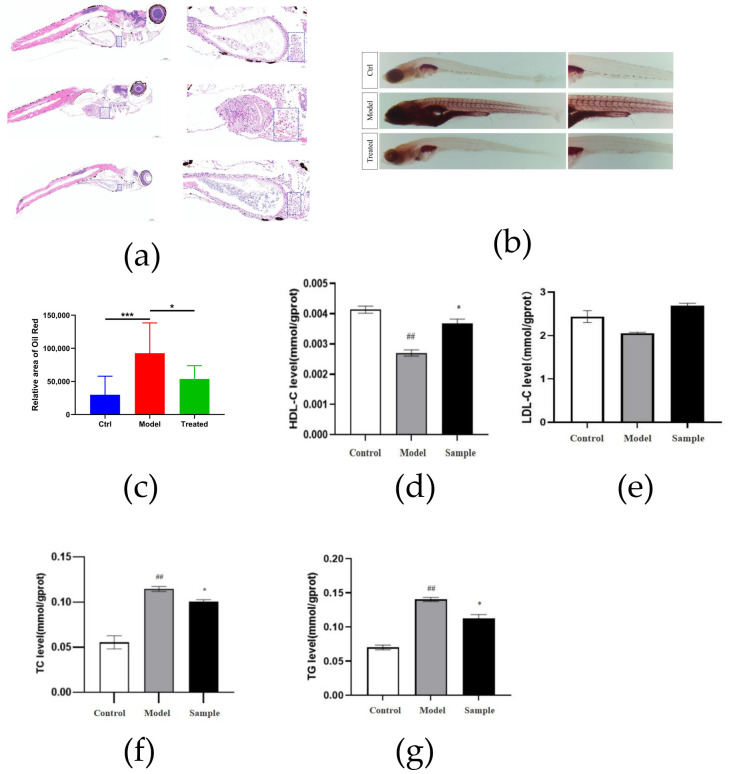
A001-A-08 attenuates high-fat-diet-induced lipid accumulation and lipid-index disturbance in zebrafish. (**a**) H&E-stained sagittal sections and liver regions. (**b**) Representative Oil Red O staining. (**c**) Quantification of Oil Red O-positive area. (**d**–**g**) HDL-C, LDL-C, TG, and TC levels. Data are presented as mean ± SD. Asterisks indicate significant differences between the indicated groups (* *p* < 0.05, *** *p* < 0.001). Control, normally fed zebrafish larvae; Model, high-fat-diet-induced lipid metabolism disorder model; Sample/Treated/A001-A-08, high-fat-diet-induced zebrafish treated with A001-A-08, number signs indicate significant differences compared with the control group (## *p* < 0.01).

H&E staining showed that high-fat feeding induced hepatic enlargement and vacuolar-like structural changes compared with the control group (Figure 2a). A001-A-08 treatment partially alleviated these histological abnormalities, with less pronounced hepatic vacuolation and improved tissue morphology. Oil Red O staining further confirmed that the model group exhibited extensive neutral lipid accumulation, especially in the abdominal and visceral regions (Figure 2b). Quantification showed that the Oil Red O-positive area was significantly increased by high-fat feeding and decreased after A001-A-08 intervention (Figure 2c). The relatively large variation observed in the model group may reflect individual differences in larval food intake, lipid absorption, and sensitivity to high-fat challenge, which are common in diet-induced zebrafish lipid-accumulation models.

The biochemical lipid indices were consistent with the staining results. HDL-C decreased in the model group and increased after A001-A-08 intervention (Figure 2d). LDL-C showed a limited or less consistent response compared with the other lipid indices (Figure 2e), suggesting that the intervention did not uniformly modify all lipid fractions. TG and TC were increased in the high-fat model and reduced after A001-A-08 treatment (Figure 2f,g). Together, the histological, staining, and biochemical results demonstrate a lipid-lowering phenotype in high-fat zebrafish.

### 3.3. Transcriptomic Quality Control and Differential Expression Patterns After A001-A-08 Intervention

**Figure 3:**
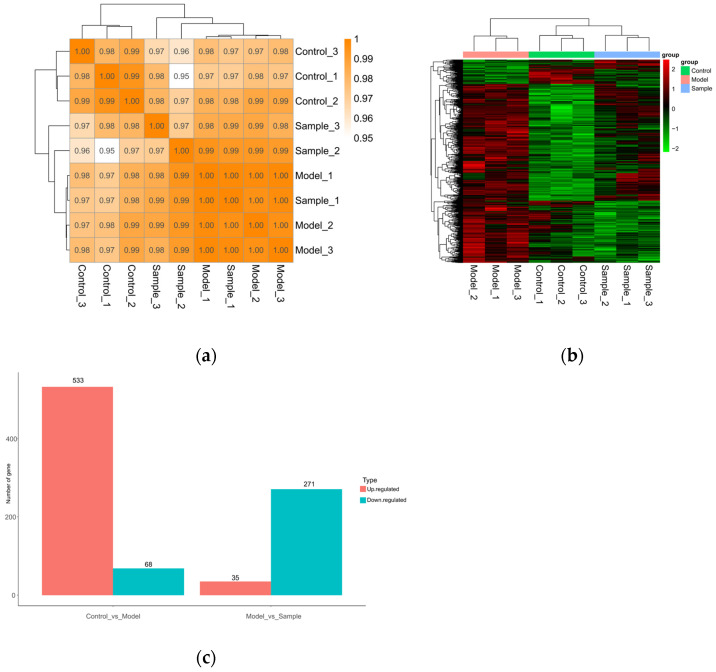
Transcriptomic quality control and differential expression patterns after A001-A-08 intervention. (**a**) Sample correlation heatmap. (**b**) Differential gene clustering heatmap. (**c**) Differential gene counts.

Transcriptomic quality and sample-relatedness analysis showed high within-group consistency, with correlation coefficients mostly close to 1.00 (Figure 3a). Hierarchical clustering of differentially expressed genes separated the control, model, and A001-A-08 groups (Figure 3b). Differential gene counts indicated that high-fat feeding and A001-A-08 exposure both altered host gene expression profiles (Figure 3c). Additional transcriptomic outputs, including PCA, expression trend analysis, genomic distribution, volcano plots, and GO/KEGG enrichment profiles, are shown in Figure S2.

### 3.4. Transcriptomic Network and Enrichment Analyses Highlighted Lipid Transport and Metabolic Remodeling

**Figure 4:**
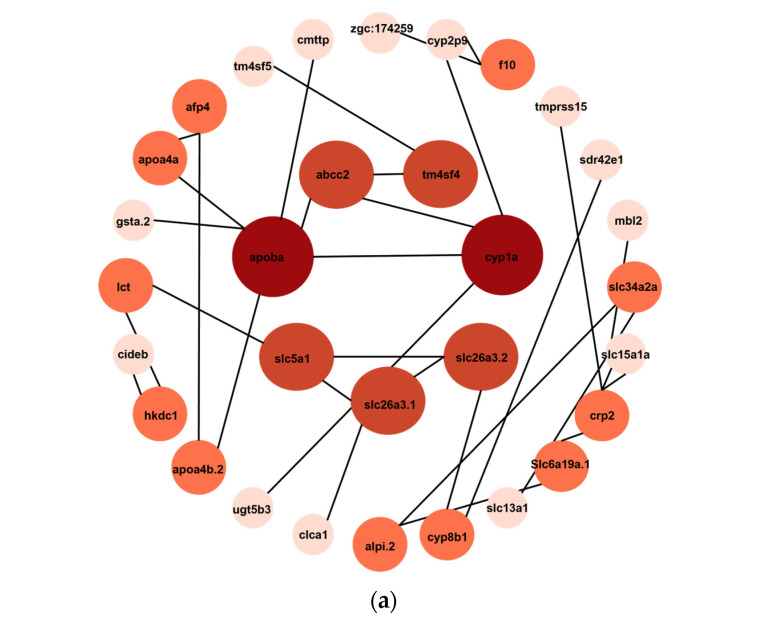

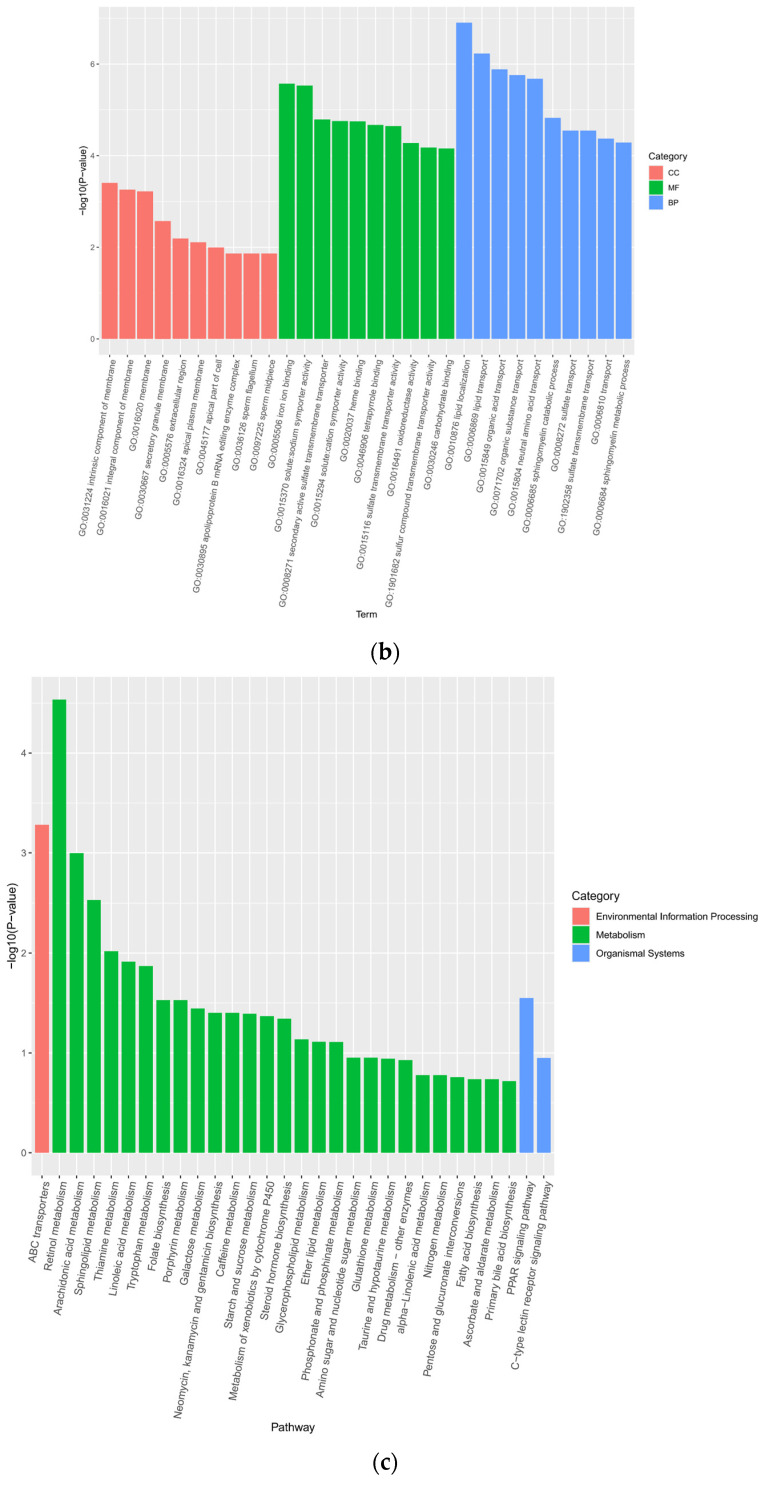
(**a**) Transcriptomic network and functional enrichment analyses of lipid metabolism-related responses to A001-A-08. (**b**) GO enrichment in the model-versus-sample comparison. (**c**) KEGG enrichment in the model-versus-sample comparison.

The protein–protein interaction network of selected lipid metabolism-related genes placed *apoba*, *cyp1a*, *abcc2*, *slc5a1*, *tmafs4*, and several transport or lipid-related genes near the center of the network (Figure 4a). GO enrichment for the model-vs-sample comparison involved membrane components, transporter activity, fatty acid transport, lipid transporter activity, and related biological processes (Figure 4b). KEGG enrichment emphasized ABC transporters, arachidonic acid metabolism, sphingolipid metabolism, steroid hormone biosynthesis, linoleic acid metabolism, and glycerophospholipid metabolism (Figure 4c). These pathway changes were consistent with the observed lipid phenotype and suggested that lipid transport, membrane transport, and lipid-related metabolic processes were associated with the A001-A-08 response.

### 3.5. Broad-Coverage Metabolomics Supported Systemic Metabolic Remodeling After A001-A-08 Intervention

**Figure 5:**
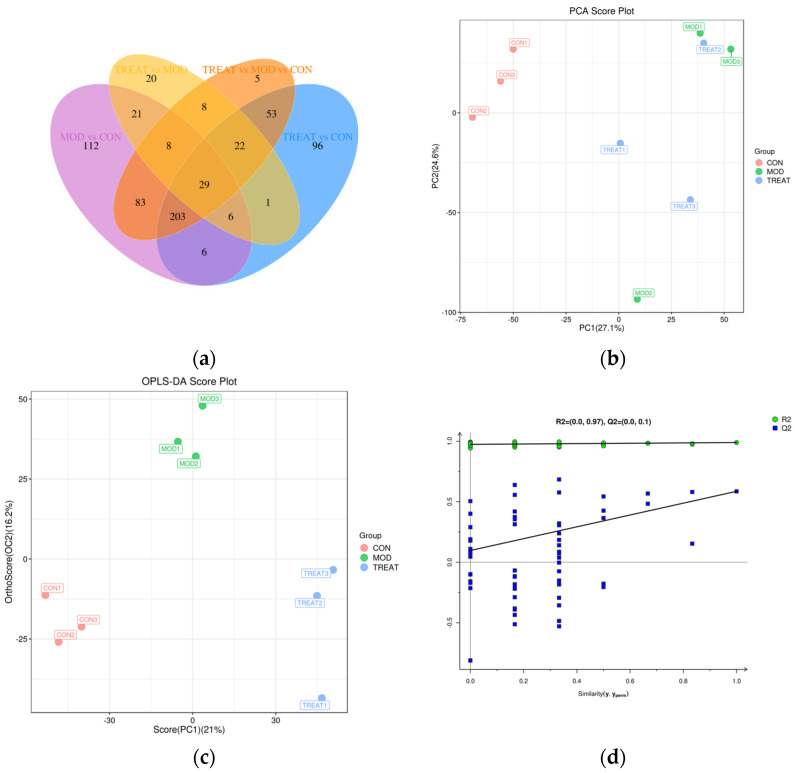

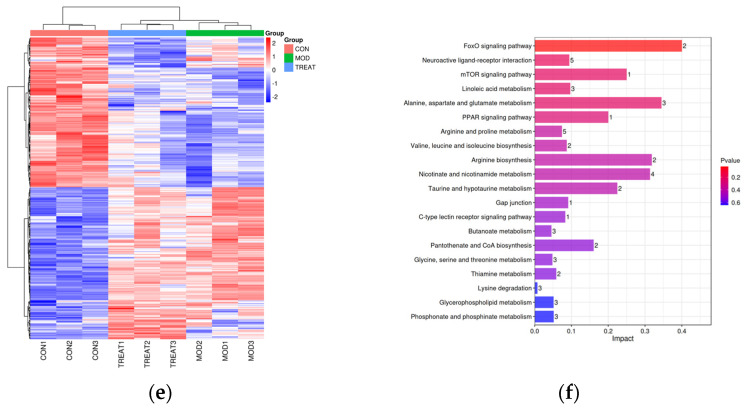
Broad-coverage LC-MS metabolomic profiling after A001-A-08 intervention. (**a**) Venn diagram of MS/MS-level differential metabolites. (**b**) PCA score plot. (**c**) OPLS-DA score plot. (**d**) OPLS-DA permutation test. (**e**) Heatmap of differential metabolites. (**f**) KEGG pathway mapping of differential metabolites.

The metabolomic Venn analysis showed that the three pairwise comparisons shared 29 annotated MS/MS-level differential metabolites, while each comparison also contained unique features (Figure 5a). PCA separated the control, model, and A001-A-08 groups, indicating global metabolic differences among treatments (Figure 5b). The OPLS-DA score plot further separated comparison groups, and the permutation test did not show an obvious overfitting pattern in the displayed model (Figure 5c,d). Additional metabolomic outputs, including metabolite-overlap analysis, global heatmap, differential-feature visualization, base peak chromatograms, and quality-control plots, are provided in Figure S3.

The heatmap of differential metabolites showed that high-fat feeding and A001-A-08 intervention reshaped metabolite abundance patterns across samples (Figure 5e). KEGG pathway mapping highlighted amino acid metabolism, nicotinate and nicotinamide metabolism, glycerophospholipid metabolism, and linoleic acid metabolism (Figure 5f). These pathway signals were interpreted together with transcriptomic and qRT-PCR results, particularly changes related to glycerophospholipid metabolism, linoleic acid metabolism, nicotinate/nicotinamide metabolism, and lipid-transport pathways. This cross-dataset overlap suggests that A001-A-08 intervention was associated with coordinated host metabolic remodeling rather than isolated metabolite changes.

### 3.6. Representative Metabolite Features Reflected Treatment-Associated Metabolic Shifts

**Figure 6:**
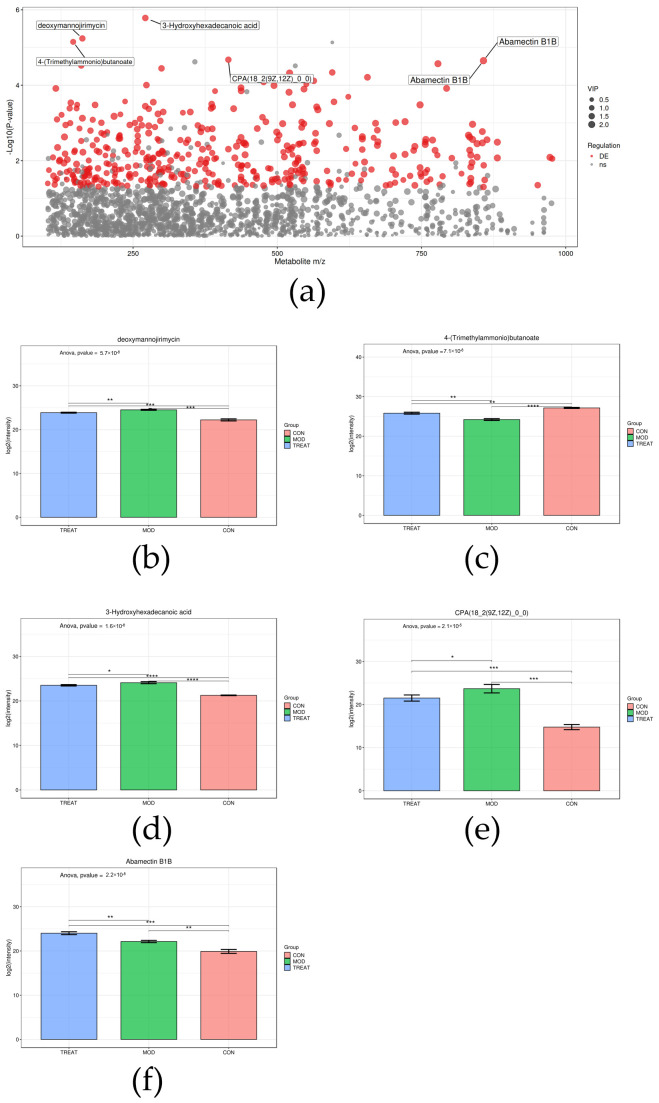
Representative annotated metabolite features responsive to high-fat feeding and A001-A-08 intervention. (**a**) Differential metabolite feature plot. (**b**–**f**) Representative abundance patterns of selected annotated metabolite features. Data in metabolite abundance panels are presented as mean ± SD. Asterisks indicate significant differences between the indicated groups (* *p* < 0.05, ** *p* < 0.01, *** *p* < 0.001, **** *p* < 0.0001). ns, not significant.

Several annotated features with differential abundance were displayed as representative metabolite-level responses (Figure 6a–f). Deoxymannojirimycin, 4-(trimethylammonio) butanoate, 3-hydroxyhexadecanoic acid, CPA (18:2(9Z,12Z) _0_0), alpha-glutamyltryptophan, and anserine showed treatment-associated abundance patterns, indicating that A001-A-08 exposure was accompanied by changes in specific metabolite features.

Several pathways highlighted by the metabolomic analysis overlapped conceptually with the transcriptomic results, including glycerophospholipid metabolism, linoleic acid metabolism, and signaling pathways linked to lipid homeostasis. Such overlap strengthens the interpretation that A001-A-08 affected host lipid-related metabolism at multiple biological levels.

### 3.7. A001-A-08 Altered Gut Microbial Diversity and Community Structure

**Figure 7:**
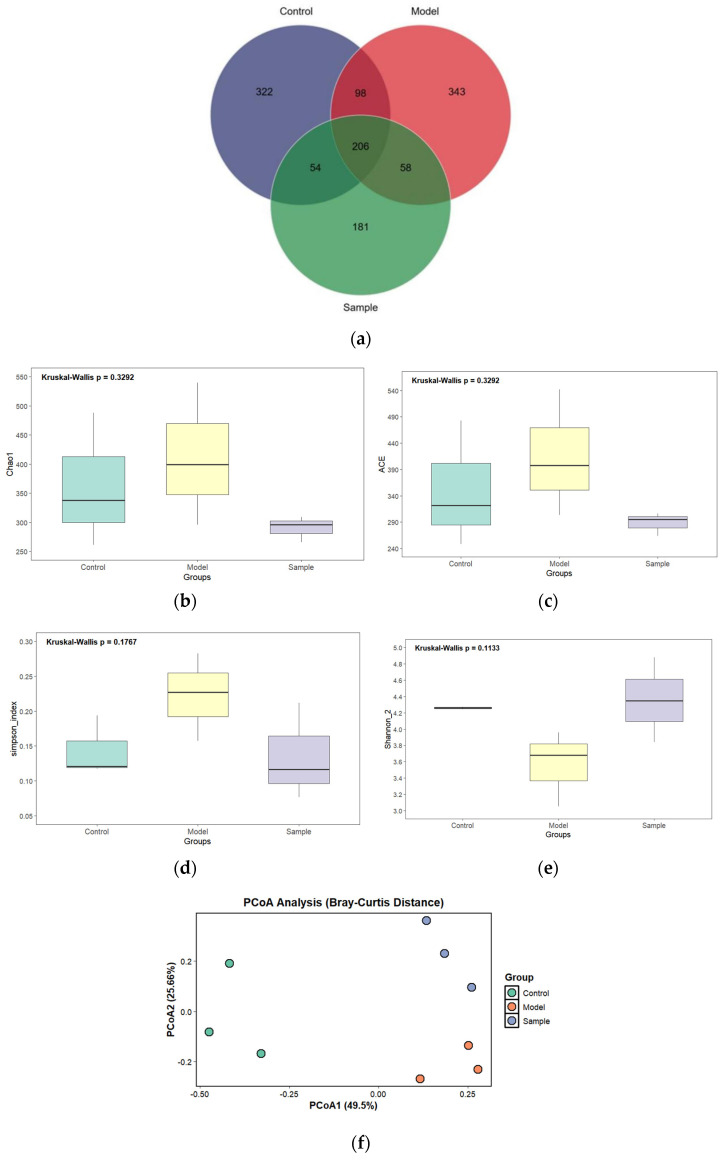
Gut microbiota diversity and community structure after A001-A-08 intervention. (**a**) ASV overlap. (**b**–**e**) Richness and alpha-diversity-related analyses. (**f**) PCoA based on Bray–Curtis distance.

16S rRNA gene amplicon sequencing showed that the control, model, and A001-A-08 groups shared a core set of ASVs, while each group also contained unique ASVs (Figure 7a). The rarefaction and richness-related analyses supported adequate sequencing depth and showed differences in community richness and diversity among groups (Figure 7b–e). PCoA based on Bray–Curtis distance indicated that the model group shifted away from the control group, whereas the A001-A-08 group occupied an intermediate or partially distinct position (Figure 7f).

### 3.8. A001-A-08 Modulated High-Fat-Associated Gut Microbiota Composition and Microbial Biomarkers

**Figure 8:**
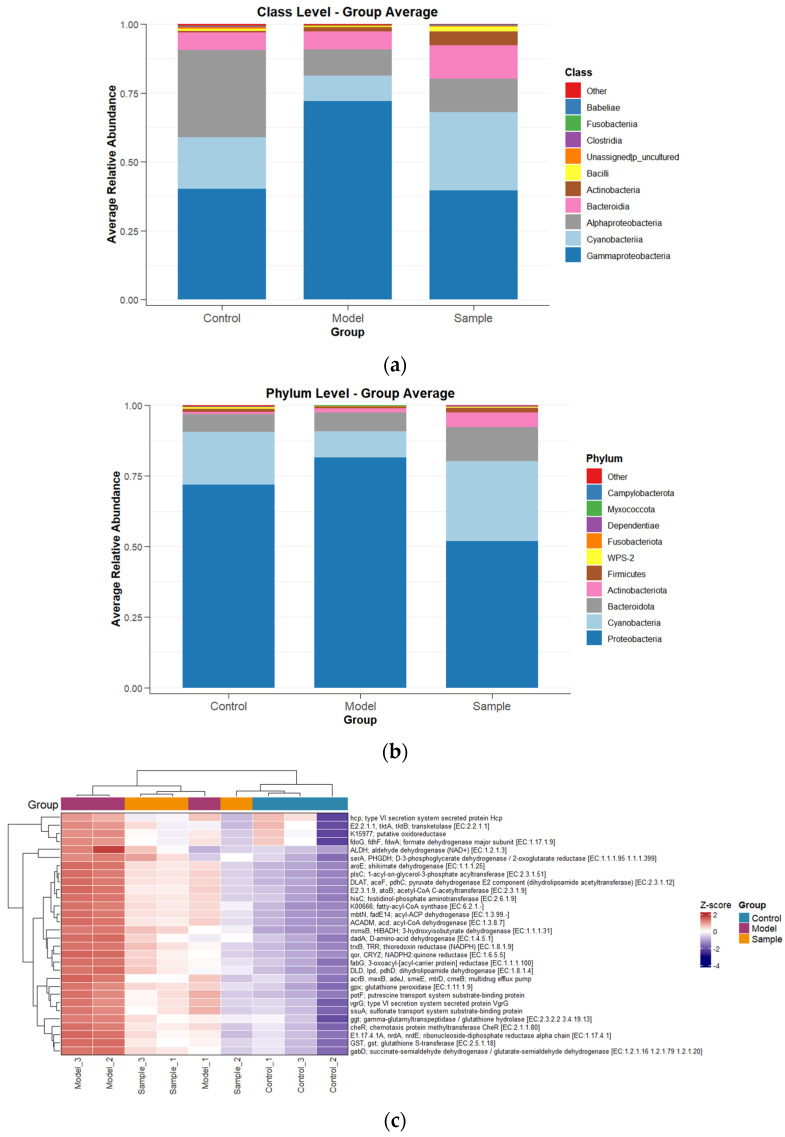
Gut microbiota composition and differential microbial features after A001-A-08 intervention. (**a**) Phylum-level composition (**b**) Genus-level composition. (**c**) Differential microbial features.

At the phylum level, *Proteobacteria* dominated the zebrafish gut microbiota, and its relative contribution differed among groups. Because *Proteobacteria* expansion is often considered a microbial signature of diet-induced dysbiosis or intestinal ecological disturbance, the partial shift after A001-A-08 treatment may reflect an association between microbial restructuring and improved lipid phenotype (Figure 8a). At the genus level, the model group showed changes in genera such as *Pseudomonas*, *Enterobacter*, *Microbacterium*, *Bosea*, *Lactobacillus*, and *Bifidobacterium* (Figure 8b). The LEfSe heatmap and biomarker analysis identified taxa that discriminated among the experimental groups (Figure 8c and Figure S2).

### 3.9. qRT-PCR Validated Down-Regulation of Lipid Metabolism- and Transport-Related Genes

**Figure 9:**
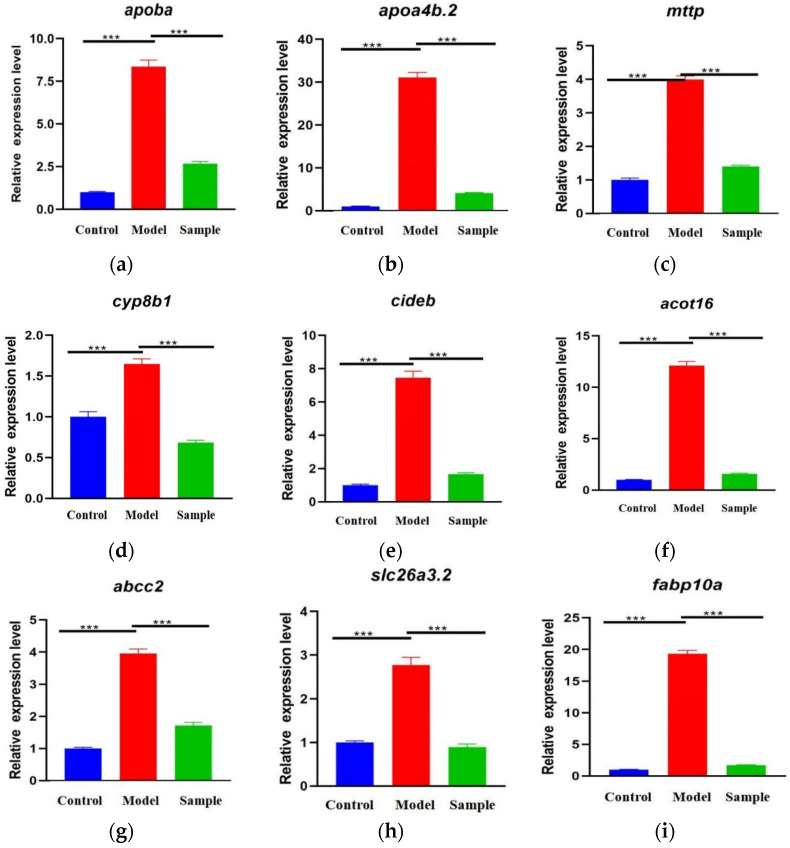
qRT-PCR validation of lipid metabolism- and transport-related genes. (**a**–**i**) Relative expression of *apoba*, *apoa4b.2*, *mttp*, *cyp8b1*, *cideb*, *acot16*, *abcc2*, *slc26a3.2*, and *fabp10a*. Data are presented as mean ± SD. Asterisks indicate significant differences between the indicated groups (*** *p* < 0.001).

qRT-PCR validation showed that the high-fat model markedly increased the expression of lipid metabolism- and transport-related genes, including *apoba*, *apoa4b.2*, *mttp*, *cyp8b1*, *cideb*, *acot16*, *abcc2*, *slc26a3.2*, and *fabp10a* (Figure 9a–i). A001-A-08 intervention significantly reduced the expression of all nine genes compared with the model group. The strongest treatment-associated decreases were observed for genes related to apolipoprotein assembly, lipid transport, bile acid-related metabolism, and fatty acid binding or intracellular trafficking.

## 4. Discussion

A001-A-08 was isolated from Rushan acid whey, a by-product of a traditional fermented dairy food. The strain showed in vitro cholesterol removal, BSH activity, gastrointestinal tolerance, auto-aggregation, γ-hemolysis, and acceptable survival in the zebrafish working environment. These characteristics match the early-stage requirements for evaluating candidate probiotic LAB [5,6]. However, they do not substitute for genome-level safety assessment, transferable antibiotic-resistance evaluation, or product-matrix validation [32,33].

The in vitro cholesterol-lowering profile of A001-A-08 is biologically plausible. BSH-producing lactobacilli can alter bile salt metabolism, potentially affecting cholesterol utilization and bile acid cycling [9,10]. Recent studies also support links between LAB intervention, gut microbial remodeling, bile acid metabolism, and lipid improvement in high-fat models [12,34]. Related LAB interventions have improved lipid metabolism through microbiota or fermented-food matrices in rodent models [35,36,37]. Similar effects have also been reported for other LAB strains in high-fat models [38,39]. However, cholesterol removal and BSH activity are screening endpoints rather than definitive mechanisms. Therefore, the present study combined in vitro selection with an in vivo zebrafish model. The reduction in Oil Red O staining, lower TC and TG, increased HDL-C, and improved hepatic morphology collectively support a treatment-associated lipid-lowering effect.

The zebrafish data are relevant for functional-food strain screening. Zebrafish larvae enable rapid visualization of lipid accumulation and are increasingly used for evaluating lipid metabolism and hepatic injury [14,15,16]. They are also useful for monitoring safety-related endpoints and high-throughput toxicity responses [40]. Nutritionally induced zebrafish models show conserved lipid-distribution and transcriptomic responses to high-fat or high-cholesterol challenges [41,42]. Broader probiotic application and encapsulation reviews also highlight storage viability, matrix compatibility, and delivery efficiency as key variables for future product development [4,43,44].

Transcriptomic and qRT-PCR data provided associative molecular context for the phenotype rather than definitive mechanistic proof. High-fat feeding activated genes involved in lipoprotein assembly, bile acid-related metabolism, fatty acid binding, and lipid transport, whereas A001-A-08 reduced the expression of these genes. The down-regulation of apoba and mttp suggests less pressure on ApoB-containing lipoprotein assembly [45,46]. Changes in *cyp8b1* and *abcc2* may reflect modulation of bile acid synthesis and transport [47,48]. The decreases in *cideb* and *fabp10a* are also consistent with altered lipid-droplet biology and intracellular fatty acid trafficking [49,50]. These observations require targeted mechanistic validation before a direct causal pathway can be assigned.

Integrating the transcriptomic, metabolomic, qRT-PCR, and microbiota results suggests a multi-level association between A001-A-08 exposure and lipid improvement. At the host-transcriptional level, high-fat feeding activated genes related to ApoB-containing lipoprotein assembly, lipid transport, bile acid-related metabolism, and intracellular lipid handling, whereas A001-A-08 reduced this activation. At the metabolite level, altered glycerophospholipid and linoleic-acid-related features were consistent with changes in lipid remodeling. At the microbial level, A001-A-08 partially shifted high-fat-associated gut microbial composition. Together, these data support an associative host–microbe metabolic remodeling model rather than a single direct pathway mechanism. Pathways such as glycerophospholipid metabolism, linoleic acid metabolism, PPAR signaling, FoxO signaling, and nicotinate/nicotinamide metabolism are relevant to lipid homeostasis and metabolic adaptation. Previous food-component studies have used lipidomics and transcriptomics together to explain improved hepatic lipid homeostasis [51,52].

The gut microbiota results are consistent with the broader concept that dietary lipids, probiotics, and intestinal microbial communities interact during metabolic regulation [53,54]. High-fat feeding altered the zebrafish gut microbiota, and A001-A-08 partially reshaped microbial composition. The changes in *Proteobacteria* related taxa and genera such as *Pseudomonas* and *Enterobacter* are noteworthy because *Proteobacteria* and *Enterobacteriaceae* expansion are commonly linked to dysbiosis [55,56,57]. Nevertheless, microbiota changes in this study remain associative. Antibiotic depletion, gnotobiotic zebrafish, fecal microbiota transfer, strain-specific qPCR, or metabolite supplementation would be needed to prove microbial causality.

The main contribution of this study is a preliminary chain from Rushan acid whey-derived LAB screening to zebrafish-based in vivo functional evaluation and multi-level host–microbe characterization. Unlike many strain-screening studies that stop at in vitro cholesterol removal or gastrointestinal tolerance, this work links a traditional dairy by-product-derived *L. fermentum* strain with zebrafish lipid phenotyping, host transcriptomic changes, metabolite remodeling, qRT-PCR validation, and gut microbiota profiling. The limitations are also clear. First, the study lacks whole-genome sequencing. Therefore, although hemolysis and antibiotic susceptibility provided preliminary safety information, the presence or absence of virulence genes, transferable antibiotic-resistance genes, plasmids, and mobile genetic elements could not be directly assessed. A001-A-08 should therefore be regarded as a probiotic candidate until genome-level safety evaluation is completed. Second, the strain was tested in a zebrafish bath-exposure model rather than by oral administration or incorporation into an actual fermented food matrix. Therefore, the present results should be interpreted as preliminary in vivo evidence of lipid-lowering potential, not direct proof of functional-food efficacy. Third, metabolomic and microbiota analyses were associative and require targeted and causal validation.

Overall, A001-A-08 should be regarded as a promising Rushan acid whey-derived probiotic candidate with preliminary in vitro safety-related traits and preliminary in vivo lipid-lowering evidence in zebrafish. The next steps should include whole-genome safety evaluation, survival and stability testing in Rushan-derived or other fermented dairy matrices, targeted bile acid or short-chain fatty acid profiling, and validation in a mammalian oral-administration model before any functional-food claim is made.

## 5. Conclusions

*L. fermentum* A001-A-08, isolated from Rushan acid whey, showed cholesterol-removal activity, BSH activity, gastrointestinal tolerance, auto-aggregation, gamma-hemolysis, and a mostly susceptible antibiotic profile. In high-fat-diet-induced zebrafish, the strain reduced lipid accumulation, improved hepatic morphology, lowered TC and TG, and increased HDL-C. Transcriptomic, metabolomic, qRT-PCR, and 16S rRNA sequencing analyses indicated that these effects were associated with changes in lipid transport, lipoprotein assembly, bile acid-related metabolism, selected metabolite features, and gut microbial composition. A001-A-08 is therefore a promising probiotic LAB candidate for future functional fermented-food development; however, genome-level safety assessment, mammalian oral-administration validation, and food-matrix application studies are still required before functional-food use can be established.

## Data Availability

The processed data supporting the findings of this study are included in the article and Supplementary Materials. The raw RNA-seq, metabolomics, and 16S rRNA sequencing datasets are not publicly deposited at this stage because the strain evaluated in this study is a commercially developed strain involved in an ongoing company-partnered functional-food development project, and the corresponding omics datasets are subject to company confidentiality regulations, intellectual-property protection, and patent-related procedures. The authors recognize the importance of public data deposition for reproducibility. Follow-up mouse experiments are currently being conducted to further validate the causal relationships among strain intervention, lipid metabolism pathways, and gut microbiota. After completion of these mammalian validation studies, and provided that public release does not violate applicable laws, company confidentiality regulations, or patent-protection requirements, the present datasets will be deposited together with the mouse-validation datasets in appropriate public repositories. Additional data may be made available from the corresponding author upon reasonable request, where permitted by institutional, legal, and company confidentiality requirements.

## Supplementary Materials

The following supporting information can be downloaded at: https://www.mdpi.com/article/10.3390/foods15142536/s1, Figure S1: Additional in vitro screening outputs for Rushan-derived LAB isolates, including the cholesterol standard curve, cell-surface hydrophobicity, and complete hemolysis-related plates. Data in quantitative panels are presented as mean ± SD. Different lowercase letters above bars indicate significant differences among strains (*p* < 0.05); Figure S2: Additional transcriptomic outputs supporting Figure 3, including PCA, expression trend analysis, genomic distribution, volcano plots, and GO/KEGG enrichment profiles; Figure S3: Additional metabolomic outputs supporting Figure 4 and Figure 5, including metabolite-overlap analysis, global heatmap, differential-feature visualization, base peak chromatograms, and quality-control plots; Table S1: Bacterial strains used in this study; Table S2. Main reagents; Table S3: Main instruments and equipment; Table S4: Composition of MRS broth; Table S5: E3 medium preparation; Table S6: Viability of freeze-dried A001-A-08 powder in E3 medium; Table S7: Acid tolerance of Rushan-derived lactic acid bacteria under different pH conditions, Values are presented as mean ± SD (n = 3). Different lowercase letters within the same column indicate significant differences among strains (*p* < 0.05); different uppercase letters within the same row indicate significant differences among treatments (*p* < 0.05). “--” indicates not detected; Table S8: Bile salt tolerance of Rushan-derived lactic acid bacteria under different bile salt concentrations, Values are presented as mean ± SD (n = 3). Different lowercase letters within the same column indicate significant differences among strains (*p* < 0.05); different uppercase letters within the same row indicate significant differences among treatments (*p* < 0.05); Table S9: Tolerance of Rushan-derived lactic acid bacteria in simulated gastrointestinal fluids, Values are presented as mean ± SD (n = 3). Different lowercase letters within the same column indicate significant differences among strains (*p* < 0.05); different uppercase letters within the same row indicate significant differences among treatments (*p* < 0.05); Table S10: Auto-aggregation ability of Rushan-derived lactic acid bacteria at different incubation times, Values are presented as mean ± SD (n = 3). Different lowercase letters within the same column indicate significant differences among strains (*p* < 0.05); different uppercase letters within the same row indicate significant differences among time points (*p* < 0.05); Table S11: Antibiotic susceptibility of Rushan-derived lactic acid bacteria, Antibiotic susceptibility was interpreted according to Clinical and Laboratory Standards Institute (CLSI) criteria. S, susceptible; I, intermediate; R, resistant.

## Abbreviations

16S rRNA	16S ribosomal RNA
ASV	Amplicon sequence variant
BSH	Bile salt hydrolase
CFU	Colony-forming unit
CLSI	Clinical and Laboratory Standards Institute
CM-DiI	Chloromethylbenzamido derivative of DiI
DESeq2	Differential expression analysis for sequence count data 2
GO	Gene Ontology
H&E	Hematoxylin and eosin
HDL-C	High-density lipoprotein cholesterol
HMDB	Human Metabolome Database
KEGG	Kyoto Encyclopedia of Genes and Genomes
LAB	Lactic acid bacteria
LC-MS	Liquid chromatography–mass spectrometry
LDL-C	Low-density lipoprotein cholesterol
LEfSe	Linear discriminant analysis effect size
MRS	De Man, Rogosa and Sharpe
MS/MS	Tandem mass spectrometry
OD	Optical density
OPLS-DA	Orthogonal partial least-squares discriminant analysis
PCA	Principal component analysis
PCoA	Principal coordinate analysis
PICRUSt2	Phylogenetic Investigation of Communities by Reconstruction of Unobserved States 2
qRT-PCR	Quantitative reverse-transcription polymerase chain reaction
RNA	Ribonucleic acid
SD	Standard deviation
TC	Total cholesterol
TG	Triglyceride
XCMS	Various forms of chromatography mass spectrometry
