# Supplementary material for: A Rushan Acid Whey-Derived Limosilactobacillus fermentum A001-A-08 Attenuates Lipid Accumulation in High-Fat-Diet-Induced Zebrafish
Source: Foods. 2026 Jul 17;15(14):2536. doi: 10.3390/foods15142536 (PMC13409735; doi:10.3390/foods15142536)
Supplement: Supplementary file 1 [file foods-15-02536-s001.zip › foods-4416714-supplementary.pdf]

Supplementary Materials

Figure S1.

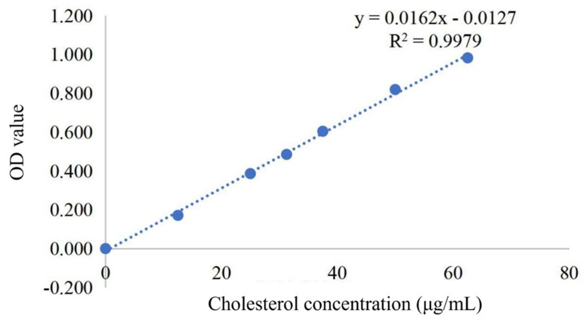

(a)

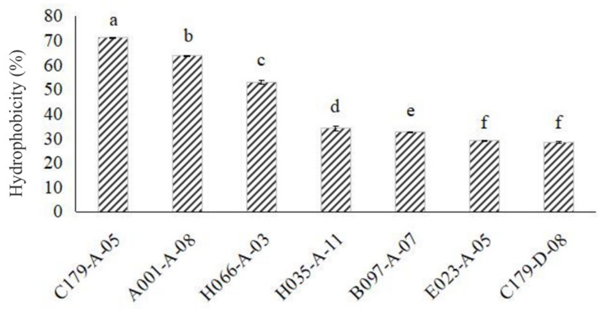

(b)

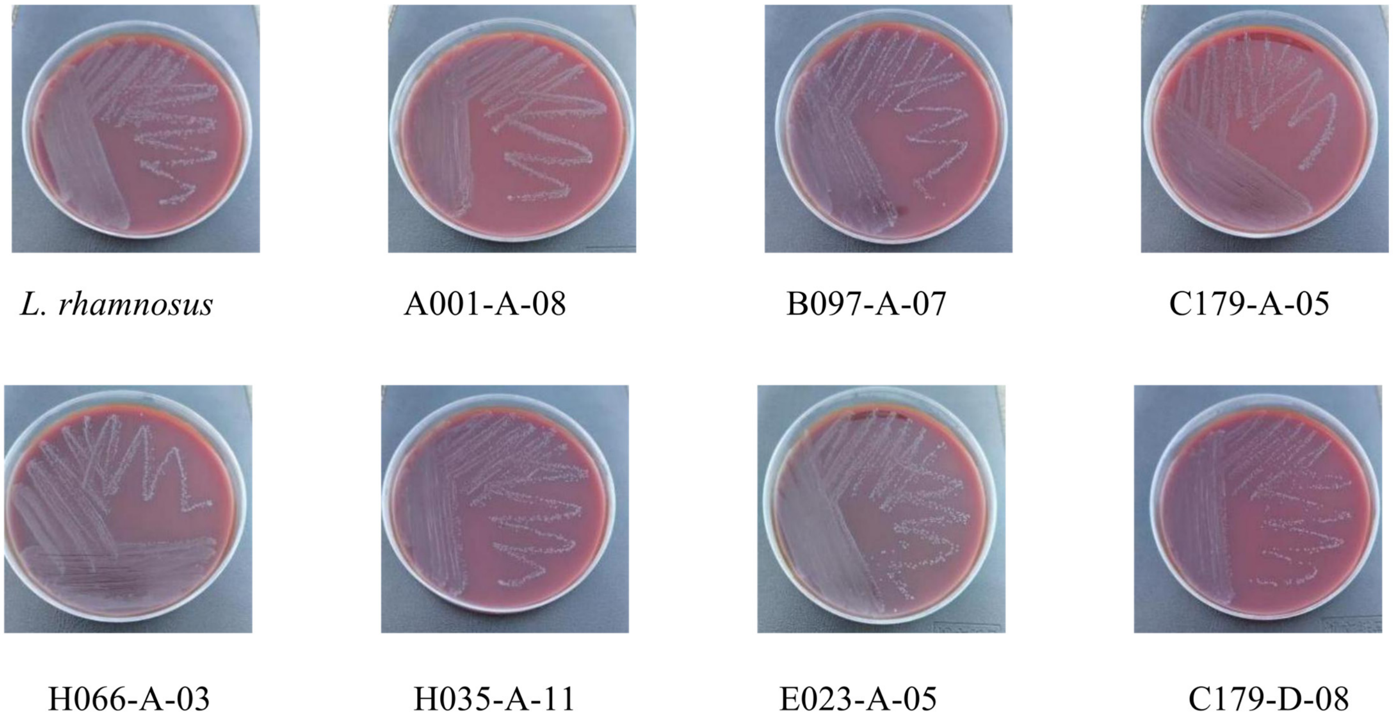

(c)

Figure S1. Additional in vitro screening outputs for Rushan-derived LAB isolates, including the cholesterol standard curve, cell-surface hydrophobicity, and complete hemolysis-related plates. Data in quantitative panels are presented as mean ± SD. Different lowercase letters above bars indicate significant differences among strains ($p < 0.05$).

Figure S2.

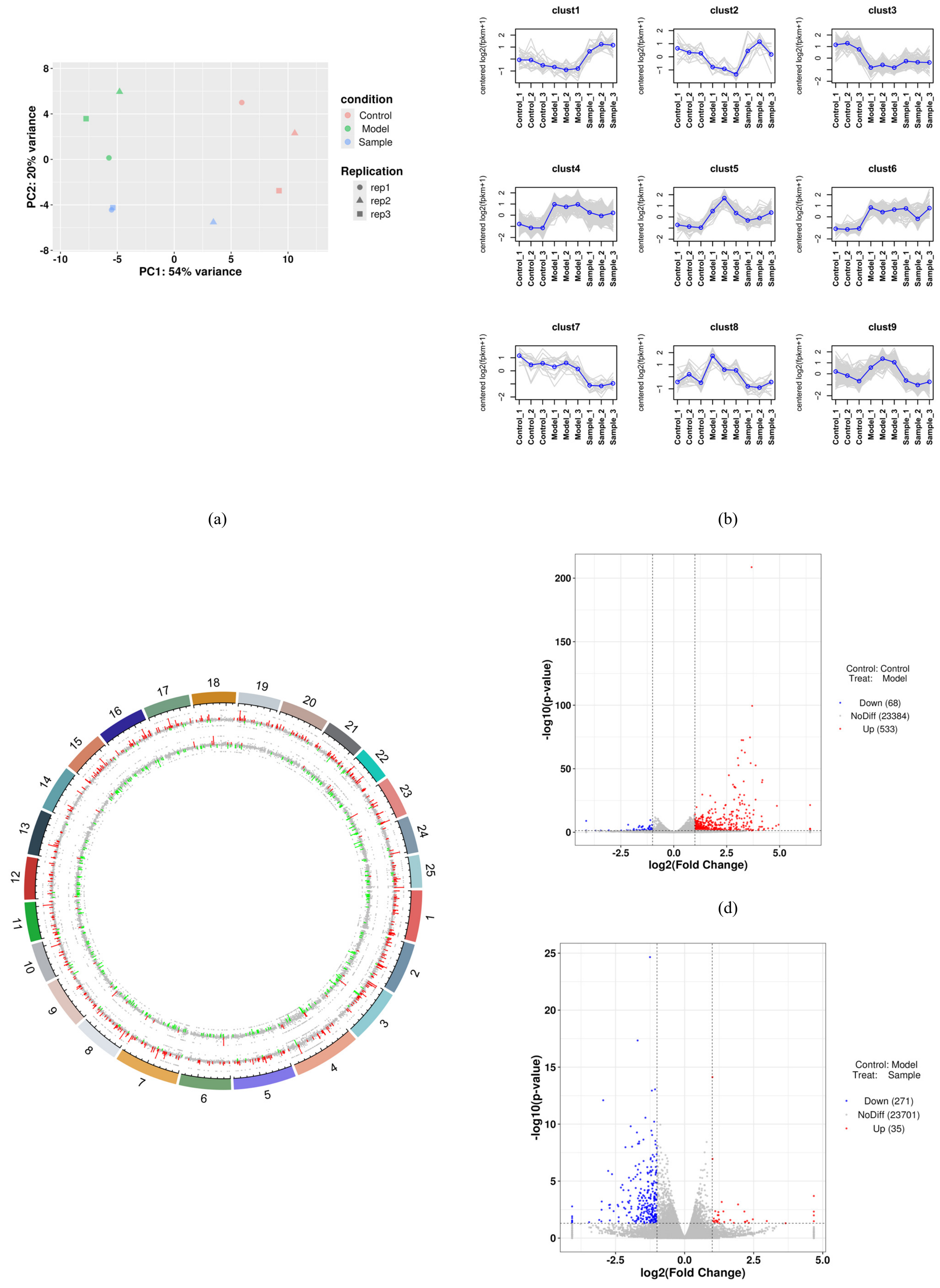

(a) (b)

(c) (d) (e)

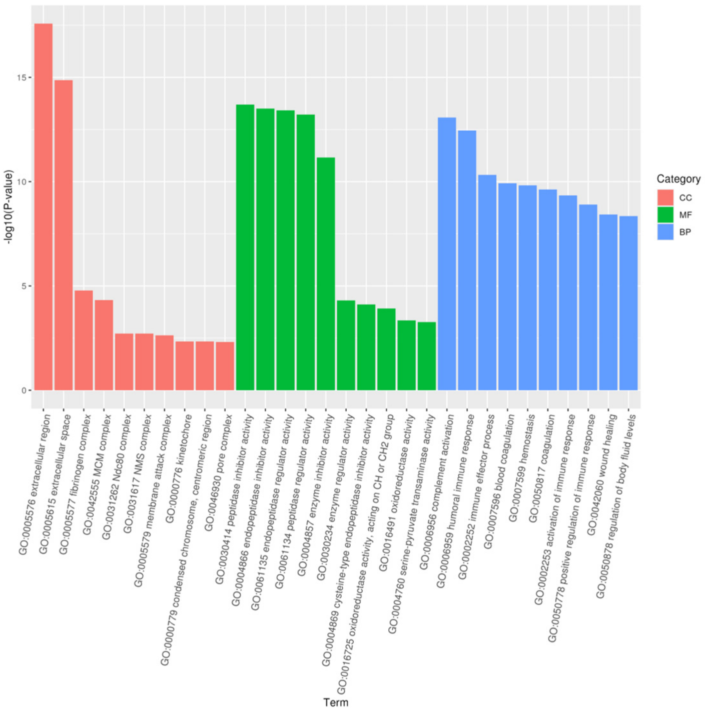

(f)

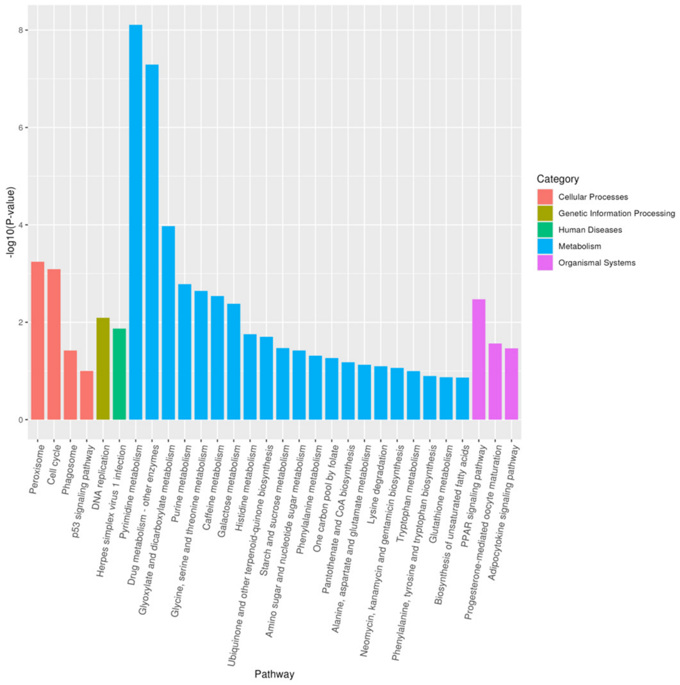

(g)

Figure S2. Additional transcriptomic outputs supporting Figure 3, including PCA, expression trend analysis, genomic distribution, volcano plots, and GO/KEGG enrichment profiles.

Figure S3.

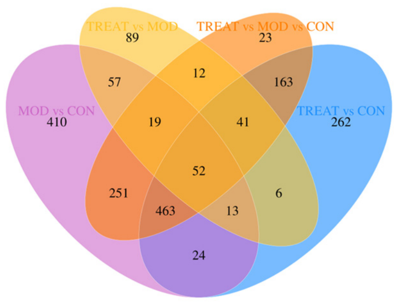

(a)

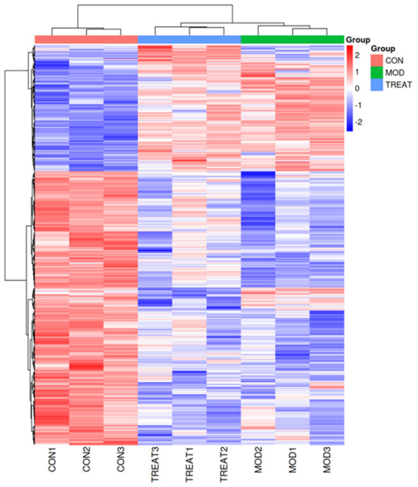

(b)

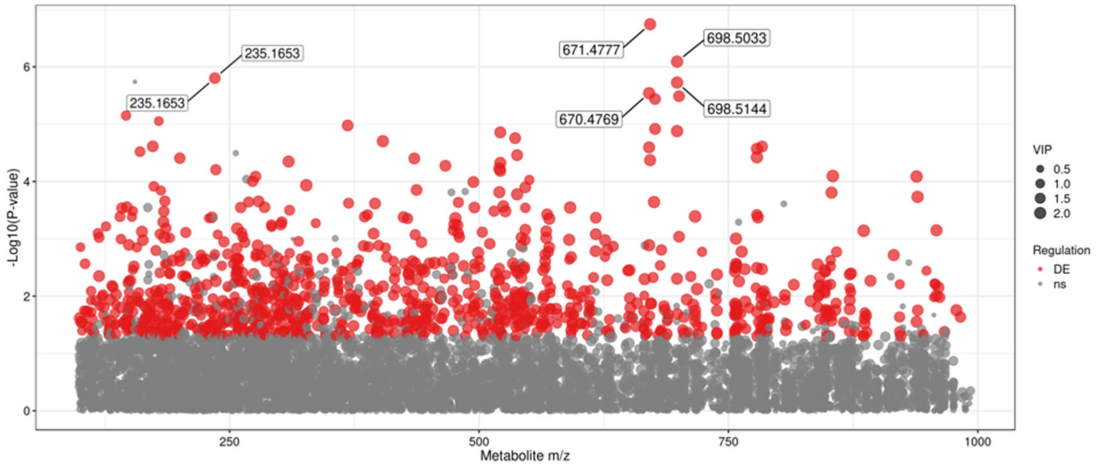

(c)

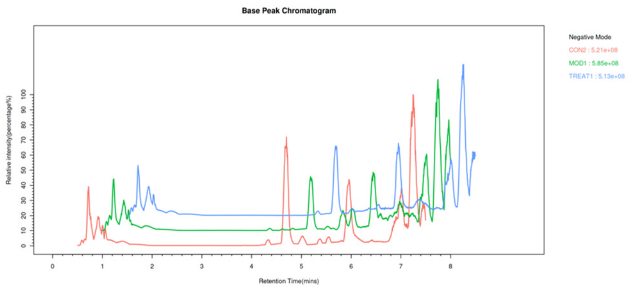

(d)

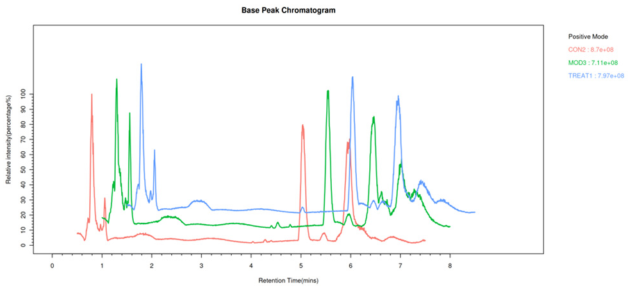

(e)

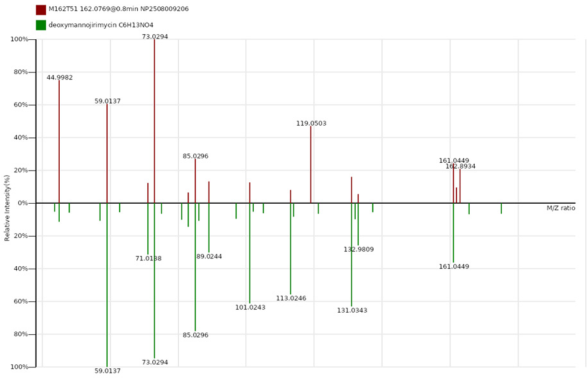

(f)

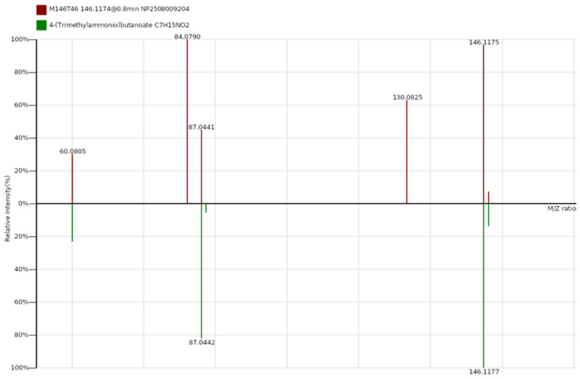

(g)

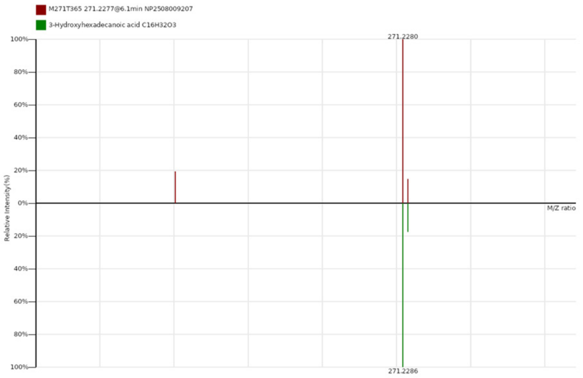

(h)

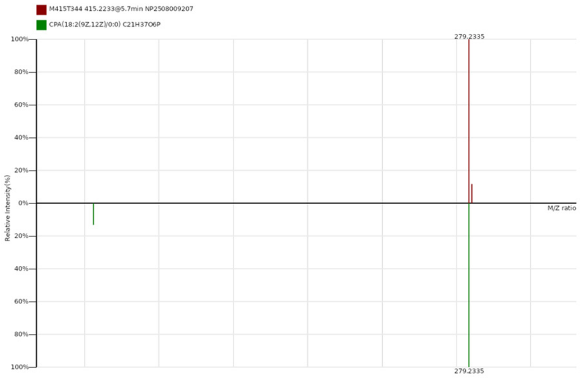

(i)

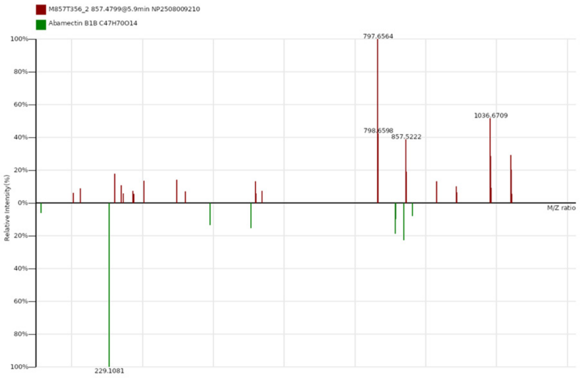

(j)

Figure S3. Additional metabolomic outputs supporting Figures 4 and 5, including metabolite-overlap analysis, global heatmap, differential-feature visualization, base peak chromatograms, and quality-control plots.

Table S1. Bacterial strains used in this study

Strain code	Species
E023-A-05	*Lactiplantibacillus plantarum*
A001-A-08	*Limosilactobacillus fermentum*
B097-A-07	*Lacticaseibacillus paracasei*
C179-A-05	*Lactiplantibacillus plantarum*
C179-D-08	*Lactiplantibacillus plantarum*
H035-A-11	*Lacticaseibacillus paracasei*
C133-B-14	*Enterococcus faecium*
D158-A-04	*Enterococcus durans*
H066-A-03	*Lacticaseibacillus paracasei*

Table S2. Main reagents

Reagent	Catalog No.	Manufacturer
High-density lipoprotein cholesterol (HDL-C) assay kit	A112-1-1	Nanjing Jiancheng Bioengineering Institute
Low-density lipoprotein cholesterol (LDL-C) assay kit	A113-1-1	Nanjing Jiancheng Bioengineering Institute
Total cholesterol (TC) assay kit	A111-1-1	Nanjing Jiancheng Bioengineering Institute
Triglyceride (TG) assay kit	A110-2-1	Nanjing Jiancheng Bioengineering Institute
Oil Red O staining kit	GP1067	Servicebio Technology Co., Ltd. (Wuhan, China)

Table S3. Main instruments and equipment

Instrument	Model	Manufacturer
Inverted microscope	CKX41	Olympus, Japan
Ultra-low-temperature freezer	MDF-382E(N)	Sanyo, Japan
Analytical balance	BT224S	Sartorius, Germany
Digital blast drying oven	GZX-9140MBE	Shanghai Boxun Industrial Co., Ltd. Medical Equipment Factory
Desktop high-speed refrigerated centrifuge	TGL20M	Hunan Xiangli Scientific Instrument Co., Ltd.
Ultra-pure water system	DZG-303A	Shanghai Fushite Instrument Co., Ltd.

Table S4. Composition of MRS broth

Component	Amount per liter
Peptone	10 g
Yeast extract	5 g
Beef extract	10 g
Glucose	20 g
Ammonium citrate	2 g
Sodium acetate anhydrous	5 g
L-cysteine hydrochloride	0.25 g
Tween 80	1 mL
MgSO4·7H2O	0.58 g
K2HPO4	2 g
MnSO4·4H2O	0.25 g

Table S5. E3 medium preparation

Component or condition	Amount or setting
NaCl	34.8 g
KCl	1.6 g
$CaCl_2 \cdot 2H_2O$	5.8 g
$MgCl_2 \cdot 6H_2O$	9.78 g
Distilled water	Bring the final volume to 2 L
pH	Adjust to 7.2
Sterilization	Autoclave at 121 °C before use

Table S6. Viability of freeze-dried A001-A-08 powder in E3 medium

Strain code	Initial viable count in sterile water (log CFU/mL)	Initial viable count in E3 medium (log CFU/mL)	Viable count after 24 h at 28 °C in E3 medium (log CFU/mL)
A001-A-08	11.61 ± 0.01	11.44 ± 0.02	11.24 ± 0.02

Table S7. Acid tolerance of Rushan-derived lactic acid bacteria under different pH conditions

Strain code	MRS	pH 2.0	pH 2.5	pH 3.0
C133-B-14	9.72 ± 0.02 aC	--	7.88 ± 0.07 aA	9.64 ± 0.00 aB
D158-A-04	9.97 ± 0.02 bC	--	7.86 ± 0.34 abA	9.81 ± 0.03 abB
B097-A-07	10.07 ± 0.00 cA	--	10.04 ± 0.01 bcA	10.13 ± 0.05 cdA
H035-A-11	10.13 ± 0.01 cB	--	10.04 ± 0.01 bcA	10.23 ± 0.03 cdC
C179-A-05	10.24 ± 0.03 dB	--	10.03 ± 0.01 bcA	10.07 ± 0.01 dA
C179-D-08	10.26 ± 0.04 dA	--	10.27 ± 0.04 cdA	10.33 ± 0.09 bcA
E023-A-05	10.41 ± 0.01 eB	--	10.35 ± 0.01 dA	10.35 ± 0.02 cA
H066-A-03	10.62 ± 0.06 fB	9.79 ± 0.13 A	10.46 ± 0.05 deB	10.54 ± 0.03 eB
A001-A-08	10.73 ± 0.04 gA	--	10.73 ± 0.01 eA	10.80 ± 0.07 eA

Values are presented as mean ± SD (n = 3). Different lowercase letters within the same column indicate significant differences among strains ($P < 0.05$); different uppercase letters within the same row indicate significant differences among treatments ($P < 0.05$). "--" indicates not detected.

Table S8. Bile salt tolerance of Rushan-derived lactic acid bacteria under different bile salt concentrations

Strain code	MRS	0.03% bile salt	0.15% bile salt	0.30% bile salt
C133-B-14	9.69 ± 0.05aA	9.73 ± 0.13aA	9.75 ± 0.06aA	9.67 ± 0.09aA
D158-A-04	9.86 ± 0.03bA	9.77 ± 0.02aA	9.82 ± 0.07aA	9.75 ± 0.11aA
C179-A-05	10.34 ± 0.03cB	10.38 ± 0.03bB	10.10 ± 0.06bA	10.32 ± 0.03bB
H035-A-11	10.35 ± 0.05cA	10.37 ± 0.04bA	10.38 ± 0.03cA	10.39 ± 0.01bA
B097-A-07	10.39 ± 0.00cdA	10.27 ± 0.07bA	10.35 ± 0.02cA	10.35 ± 0.06bA
H066-A-03	10.50 ± 0.03deA	10.60 ± 0.03cAB	10.61 ± 0.04dB	10.62 ± 0.05cdB
C179-D-08	10.61 ± 0.01efA	10.66 ± 0.01cB	10.63 ± 0.03dAB	10.64 ± 0.01cAB
E023-A-05	10.69 ± 0.03fB	10.55 ± 0.04cA	10.64 ± 0.05dAB	10.67 ± 0.04cB
A001-A-08	10.84 ± 0.10gA	10.78 ± 0.02dA	10.78 ± 0.04eA	10.84 ± 0.02dA

Values are presented as mean ± SD (n = 3). Different lowercase letters within the same column indicate significant differences among strains ($P < 0.05$); different uppercase letters within the same row indicate significant differences among treatments ($P < 0.05$).

Table S9. Tolerance of Rushan-derived lactic acid bacteria in simulated gastrointestinal fluids

Strain code	MRS	Simulated gastric fluid, 3 h	Simulated intestinal fluid, 4 h	Simulated intestinal fluid, 8 h
E023-A-05	9.61 ± 0.05bAB	9.59 ± 0.01dA	9.68 ± 0.01eBC	9.72 ± 0.02gC
A001-A-08	9.76 ± 0.02cA	9.82 ± 0.01fB	9.73 ± 0.02eA	9.82 ± 0.02hB
B097-A-07	9.23 ± 0.02aA	9.22 ± 0.01cA	9.25 ± 0.01cA	9.21 ± 0.02cA
C179-A-05	9.64 ± 0.05bcC	9.63 ± 0.04deC	9.56 ± 0.01dB	9.45 ± 0.02dA
C179-D-08	9.67 ± 0.01bcA	9.66 ± 0.01eA	9.73 ± 0.01eB	9.67 ± 0.01fA
H035-A-11	9.35 ± 0.06aA	9.24 ± 0.01cA	9.20 ± 0.01cA	9.22 ± 0.01cA
H066-A-03	9.71 ± 0.07bcB	9.66 ± 0.02deAB	9.59 ± 0.05deAB	9.55 ± 0.01eA
C133-B-14	9.69 ± 0.06bcC	8.79 ± 0.02aAB	8.71 ± 0.01aA	8.80 ± 0.01aB
D158-A-04	9.90 ± 0.03dC	8.99 ± 0.01bB	8.91 ± 0.02bA	8.95 ± 0.03bAB

Values are presented as mean ± SD (n = 3). Different lowercase letters within the same column indicate significant differences among strains ($P < 0.05$); different uppercase letters within the same row indicate significant differences among treatments ($P < 0.05$).

Table S10. Auto-aggregation ability of Rushan-derived lactic acid bacteria at different incubation times

Strain code	2 h (%)	4 h (%)	24 h (%)	48 h (%)
E023-A-05	7.48 ± 0.19 aA	15.47 ± 0.29 bB	45.31 ± 0.16 cC	54.23 ± 0.34 bD
A001-A-08	11.22 ± 0.29 cA	18.47 ± 0.15 deB	49.76 ± 0.29 dC	64.81 ± 0.24 eD
B097-A-07	7.28 ± 0.41 aA	13.67 ± 0.40 aB	42.04 ± 0.47 bC	57.16 ± 0.25 cD
C179-A-05	8.75 ± 0.46 bA	17.05 ± 0.15 cB	57.35 ± 0.26 efC	61.43 ± 0.15 dD
C179-D-08	10.42 ± 0.47 cA	23.80 ± 0.32 fB	56.25 ± 0.49 eC	66.70 ± 0.61 fD
H035-A-11	6.70 ± 0.61 cA	15.72 ± 0.70 abcdB	32.63 ± 0.67 aC	37.39 ± 0.56 aD
H066-A-03	14.53 ± 0.76 dA	22.03 ± 0.81 efB	57.9 ± 0.36 fC	72.63 ± 0.55 gD

Values are presented as mean ± SD (n = 3). Different lowercase letters within the same column indicate significant differences among strains ($P < 0.05$); different uppercase letters within the same row indicate significant differences among time points ($P < 0.05$).

Table S11. Antibiotic susceptibility of Rushan-derived lactic acid bacteria

Antibiotic	H066-A-03	A001-A-08	B097-A-07	H035-A-11	C179-A-05	C179-D-08	E023-A-05
Teicoplanin	R	R	R	R	R	R	R
Chloramphenicol	S	S	S	S	S	S	S
Ampicillin	S	S	S	S	S	S	S
Clindamycin	S	S	S	S	S	S	S
Ciprofloxacin	S	R	I	S	R	S	R
Streptomycin	R	R	R	S	R	S	S
Tetracycline	S	S	S	S	S	S	S
Gentamicin	S	S	R	S	S	S	S
Erythromycin	S	S	S	S	S	S	S
Penicillin	S	S	S	S	S	S	S

Antibiotic susceptibility was interpreted according to Clinical and Laboratory Standards Institute (CLSI) criteria. S, susceptible; I, intermediate; R, resistant.
